# Assessment of human papillomavirus vaccination rates of adolescents in California, 2018–2019

**DOI:** 10.1016/j.pmedr.2023.102144

**Published:** 2023-02-13

**Authors:** Brooke R. Warren, Hilary Gillette-Walch, Jaime Adler, Raquel Arias, Jeffrey D. Klausner, Kimlin T. Ashing, Alessandro Villa

**Affiliations:** a School of Medicine, University of California, San Francisco. San Francisco, CA, United States; b Central California Alliance for Health. Scotts Valley, CA, United States; c Academy Health. Washington, DC, United States; d American Cancer Society. Burbank, CA, United States; e Division of Disease Prevention, Policy and Global Health, Department of Preventive Medicine, University of Southern California Keck School of Medicine. Los Angeles, CA, United States; f Division of Health Equity, City of Hope Comprehensive Cancer Center. Duarte, CA, United States; g Oral Medicine, Oral Oncology and Dentistry, Miami Cancer Institute. Miami, FL, United States; h Herbert Wertheim College of Medicine, Florida International University. Miami, FL, United States

**Keywords:** HPV vaccination, Cancer prevention, Vaccinations, Adolescents

## Abstract

Accurate documentation of state-level human papillomavirus (HPV) vaccination is required for public health planning and to inform corrective actions. To examine the representativeness of the California Immunization Registry, we compared the National Immunization Survey (NIS)-Teen, commercial HMOs in California, Medi-Cal, and California Immunization Registry data for HPV vaccine series completion. Our objectives were to evaluate the vaccine registries, compare and report their completeness, and make recommendations on how to improve and use these studies. Vaccination values were extrapolated for all adolescents aged 13 to 17 years from 2018 to 2019 from NIS-Teen, adolescents aged 13 years from 2018 to 2019 reported in the California Immunization Registry, and adolescents aged 13 years for 2018 for commercial HMOs and Medi-Cal. HPV series completion among 13-year-olds in 2018 for commercial HMOs was 50 %, Medi-Cal was 45 %, and the California Immunization Registry was 28 %, with NIS-Teen rates for 13 to 17-year-olds at 50 % in 2018 and 54 % in 2019. Both rural and urban geographic regions were found to have low completion rates of the HPV series, with trends ranging from 13 % to 45 %. The California Immunization Registry’s lower HPV vaccine series completion among 13-year-olds compared to the other reporting sources is most likely due differences in reporting and data collection. Importantly, this data will serve as a comparator for future, similar studies of various sources of HPV vaccination rates following the passing of Bill-1797, which will mandate immunization reporting starting in January 2023.

## Highlights

• The California Immunization Registry has low HPV vaccine series completion.
• California will benefit from increased provider reporting.
• HPV vaccination in California is far below the national goal of 80 % by 13 years old.
• Limitations of the databases must be considered when making vaccination policy.

## 1 Background

Human papillomavirus (HPV) is the most commonly sexually transmitted infection in the United States. (Centers for Disease Control, 2022) Each year, persistent infection with high-risk strains of HPV causes 34,800 new cases of cervical, anal/rectal, vaginal, oropharyngeal, vulvar, and penile cancer nationally. Despite ongoing efforts to reduce the number of HPV infections, and therefore prevent HPV associated cancers with the 9-valent HPV vaccine, only 75.1 % of eligible United States adolescents aged 13 to 17 years started the HPV vaccination series in 2020, with 58.6 % up to date (Pingali et al., 2021).

The Federal Drug Administration approved the 9-valent HPV vaccine in 2014. The vaccine is routinely recommended for children aged 9 to 12, for catch-up through age 26, and for some adults aged 27 to 45. (Meites et al., 2019) The vaccine is safe and effective in preventing most HPV-related cancers and genital warts. (Johnson and Burtness, 2020, Creamer et al., 2019, Shimabukuro et al., 2019, Medeiros et al., 2020) The age of series initiation is important given that earlier vaccination is associated with both higher levels of immunogenicity and series completion. (Markowitz et al., 2014, Kavanagh et al., 2017, Beachler et al., 2016, St. Sauver et al., 2016) Therefore, the Advisory Committee on Immunization Practices (ACIP) of the United States Centers for Disease Control and Prevention (CDC) recommends vaccination for 11- to 12-year-olds, but can begin as early as 9 years. (Petrosky et al., 2015) Despite the proven effectiveness of the HPV vaccine, its uptake is consistently lower than other childhood and teenage vaccines like the Tdap and meningococcal conjugate vaccines. (Centers for Disease Control, 2022).

California has the largest population in the United States with over 39 million people located in both urban and rural regions. According to National Immunization Survey-Teen (NIS-Teen), which measures the uptake of preteen vaccines, only 52.6 % of adolescents in California aged 13 to 17 years old as of 2018 have completed the HPV vaccine series. (Centers for Disease Control, 2022, Walker et al., 2019) Studies have found that socioeconomic status, rural residence, ethnicity/race and educational policy were often strong predictors of differences in vaccine uptake, (Staples et al., 2021, Rotsides et al., 2021, Vickers et al., 2019, Perkins et al., 2016, Niccolai et al., 2011) making it essential to understand regional variation and associated correlates when assessing vaccination patterns in California.

To help understand the differences, strengths, and weaknesses of the three California vaccination surveillance sources, we compared HPV vaccination data from the CDC-sponsored NIS-Teen, quality performance metrics from public and private health plans and provider groups in California, and the statewide California Immunization Registry (CAIR). Our objectives were threefold: to evaluate the vaccine registries, to compare and report their completeness, and to make recommendations on how to improve and use these studies.

## 2 Methods

### 2.1 Data collection

#### 2.1.1 National Immunization survey (NIS)-Teen

We used the NIS-Teen for the 2018 to 2019 survey years as a data source to assess vaccine coverage levels in California. NIS-Teen is an annual, cross-sectional survey launched in 2006 to monitor vaccination coverage among adolescents 13 to 17 years of age living in the United States. (Jain et al., 2009) The NIS-Teen provides national, state, and selected local level estimates of vaccination coverage based on provider-reported vaccination histories for vaccines recommended by ACIP. HPV vaccine was added to NIS-Teen in 2008 for females and in 2012 for males. Additional details regarding NIS-Teen methodology are discussed elsewhere. (Jain et al., 2009, Wolter et al., 2017, [8]).

#### 2.1.2 Health plans and provider groups reported vaccination rates

Another data source used to assess vaccine coverage levels was from the National Committee for Quality Assurance, which collects data annually from health plans and other health care organizations. Reports were received from the California Department of Health Care Services and the Integrated Healthcare Association. The California Department of Health Care Services calculates vaccination coverage through a sample of administrative claims data from Medi-Cal managed care health plans for California adolescents with at least 11 months of continuous coverage before the data retrieval date (n = 216,618; 13-year-olds as of 2018). (Quality Measures & Reporting, 2022) The Integrated Healthcare Association reports on commercial HMO performance based on claims, encounters, and supplemental electronic health records. (Integrated Healthcare Association, 2021) In 2018, the dataset included 10 health plans (Appendix A) and 200 physician organizations (n = 101,988; 13-year-olds).

#### 2.1.3 California Immunization Registry

California’s immunization information system, the California Immunization Registry (CAIR), was another source used to assess vaccine coverage levels in California. The three California registries within CAIR are CAIR2, San Diego Immunization Registry, and Healthy Futures. CAIR2 is operated by the California Department of Public Health for 49 of the 58 California counties, the San Diego Immunization Registry operates in San Diego County, and the Healthy Futures covers 8 counties located in California’s Central Valley. To submit vaccination data, health records are sent to CAIR on each clinic’s behalf, either through EHR vendors or other third-party data submitters, using Health Level 7 Vaccine Update (HL7 VXU) messages. This data are then transmitted to CAIR via Simple Object Access Protocol web services. (Enhancing and Interoperability, 2016) As of 2019, CAIR has varying provider participation among regions. Pharmacists are the only health care providers in California who are mandated by state regulation to report immunization doses to CAIR. (California State Board of Pharmacy, 2022) Although Medi-Cal managed care plans must report to the immunization information system, a third of provider offices in 49 counties did not enter immunizations into CAIR. (Centers for Disease Control and Prevention, 2022) As of today, 2,413 distinct pediatric and family medicine sites currently submit data electronically to CAIR2. (Centers for Disease Control and Prevention, 2022).

### 2.2 Study population

Vaccination values were extrapolated from three different study populations. First, we obtained vaccination data from NIS-Teen survey for the years 2018 to 2019 for adolescents aged 13 to 17. The survey included both state and national data. For the year 2018, NIS-Teen surveyed 18,700 adolescents nationally and 356 in California. For the year 2019, NIS-Teen surveyed 18,788 adolescents nationally and 274 in California. Second, we examined data from CAIR for adolescents aged 13 for the years 2018 to 2019 (n = 558,480). Lastly, we examined data from commercial HMOs in California for adolescents aged 13 for the year 2018 (n = 101,988) and Medi-Cal (n = 400 randomly sampled from a total eligible population of 216,683 members). Individuals were included in the CAIR denominator if they met three criteria: born January 1, 2007 through December 31, 2007 (13- year-old cohort); had at least two doses of any vaccine in immunization information system; and were California residents. The year 2018 represents the most recent data on HPV vaccination for commercial HMOs and Medi-Cal, and the year 2019 represents the most recent data on HPV vaccination for CAIR.

### 2.3 Statistical analysis

Descriptive analysis was done on the data sources for HPV vaccination completion and initiation in California. Additionally, the CDC’s National Center for Health Statistics (NCHS) 2013 Urban-Rural Classification Scheme for Counties was used to analyze geographic disparities for HPV vaccine initiation and completion in California.

## 3 Results

### 3.1 Comparison of California HPV immunization databases

Table 1 shows a comparison of the statewide HPV vaccination series completion among 13-year-olds in 2018 by the three different immunization record databases. Teens enrolled in commercial HMOs in California and Medi-Cal managed care health plans had similar estimations of completion rates with 50 % and 45 %, respectively. In contrast, CAIR analyses yielded a lower completion estimate in 2018 at 28 %. Similar to the NIS-Teen findings, the proportion of California adolescents recorded in CAIR who completed the vaccine series in 2018 (28 %) was lower than the proportion who initiated it (50 %). Completion and initiation patterns followed a similar pattern in 2019 with completion at 30 % while initiation was 54 %. NIS-Teen shows that nationally in 2018, HPV series completion rates were lower among 13-year-olds versus adolescents aged 13 to 17 years with completion rates of 40 % (13-year-olds) and 51.1 % (13 to 17-year-olds). 2019 NIS-Teen data showed similar trends with 45.3 % and 54.2 % completion rates, respectively. Additionally, NIS-Teen found that while 78 % of adolescents aged 13 to 17 in California started the HPV vaccine series in 2019, only 56.4 % completed their doses (Table 2). This proportion is compared to 2018 where 74 % initiated and 52.6 % completed the series.

**Table 1:** Human papillomavirus vaccine series initiation and completion among 13-year-olds, California, 2018 and 2019.

**Data Source**	**NIS-Teen National**^a^		**CAIR**^b^		**California Commercial HMOs**^c^		**Medi-Cal**^d^	
HPV Vaccination	2018	2019	2018	2019	2018	2019	2018	2019
Initiation, % (95 % CI)			50	54				
Completion, % (95 % CI)	40 (37.0–42.9)	45.3 (42.1–48.5)	28	30	50		45	

NIS = National Immunization Survey, CAIR = California Immunization Registry, Blanks denote data not collected.

a Based on 3,927 individuals randomly sampled in the national survey who were 13 years of age at the time of the interview in 2018 and 2019.

b The 558,480 patients who turned 13 years of age in 2018 with 2 or immunizations in CAIR Were used as the denominator in this CAIR calculation.

c HPV immunization status was ascertained for all 101,988 commercial HMO 13-year-old enrollees whose 200 physician organizations in 10 health plans participate in IHA Align.Measure.Perform program.

d Based on approximately 400 patients randomly sampled for each of 53 reporting units of Medi-Cal managed care health plans from a total eligible population of 216,686 members who turned 13 years of age in 2018.

**Table 2:** Human papillomavirus vaccine series initiation and completion among 13 to 17-year-olds, NIS-Teen, 2018 and 2019.

**Data Source**	**NIS-Teen, National**^a^		**NIS-Teen, California**^b^	
HPV Vaccination	2018	2019	2018	2019
Initiation, % (95 % CI)	68.1 (66.8–69.3)	71.5 (70.1–72.8)	73.5 (96.7–79.4)	78.7 (70.6–85.1)
Completion, % (95 % CI)	51.1 (49.8–52.5)	54.2 (52.7–55.8)	52.6 (945.4–59.8)	56.4 (48.3–65)

NIS = National Immunization Survey.

a Based upon 18,788 individuals randomly sampled in the national survey ages 13 to 17 at the time of the interview in 2019. Based upon 18,700 adolescents surveyed nationally in 2018 and 18,788 adolescents in 2019.

b Based upon 356 individuals surveyed in California in 2018 and 274 in 2019.

### 3.2 HPV vaccine series completion differences among adolescent females and males

The NIS-Teen survey found that completion of HPV vaccination among 13-year-olds increased from 22.1 % in 2008 to 61.5 % in 2019 for females and 11.7 % in 2012 to 51.4 % in 2019 for males. Series completion increased for females from 50.1 % in 2018 to 61.5 % in 2019 but dropped for males from 55.1 % to 51.4 % in the same time period. Maximum population vaccination completion was 61.5 % for females and 55.4 % for males. CAIR had similar patterns of HPV vaccine series completion rates among females and males for both 2018 and 2019 with 29 % and 31 % completion for females, and 27 % and 30 % completion for males, respectively (data not shown).

### 3.3 HPV vaccine series initiation versus completion among 13-year-olds by county

In Fig. 1A, among 13-year-olds whose vaccination status is recorded in CAIR, HPV vaccine initiation ranged from 34 to 73 % by county, while vaccine completion rates ranged from 13 to 48 %. Rural and urban geographic disparities are apparent with the initiation of the HPV vaccine series being as low as 34 % in one rural county grouping and up to 73 % in an urban county (Fig. 1B). Completion of the series had similar trends with a rate as low as 13 % in one rural county and as high as 48 % in an urban county (Fig 1C). For both series initiation and completion, lower coverage was observed in the northern and eastern parts of the state .

**Fig. 1A:**
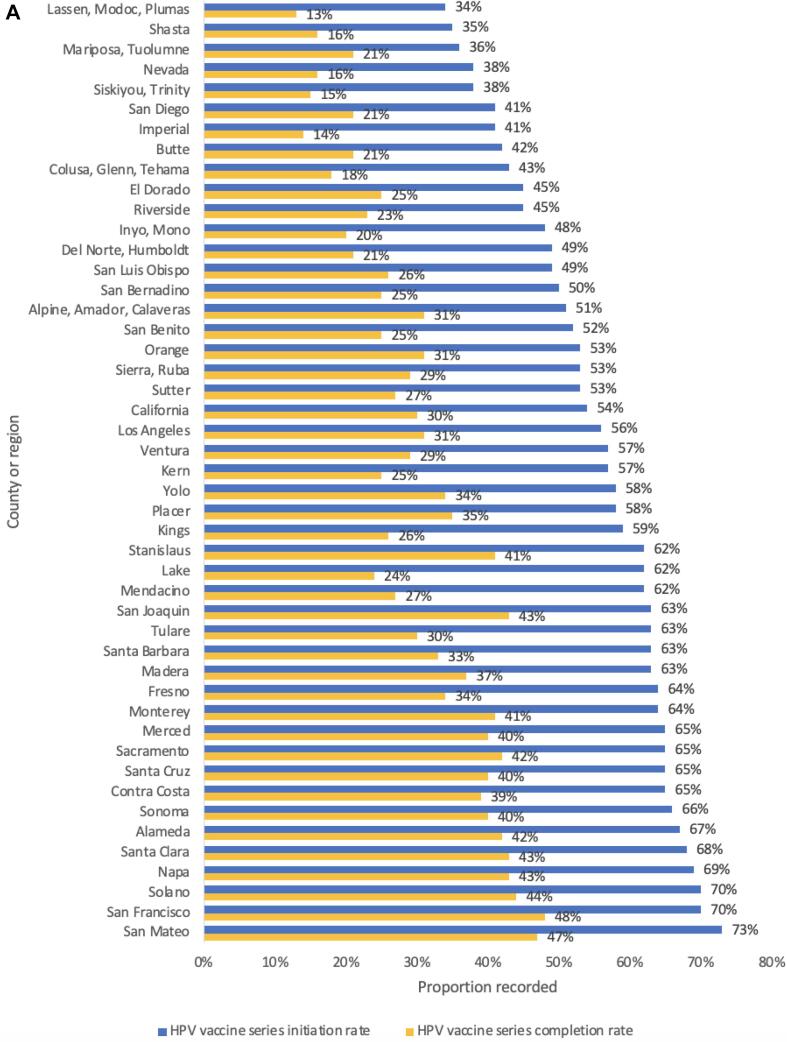
Human papillomavirus initiation and completion rate by county, California, 2019, California Immunization Registry.

**Fig. 1B:**
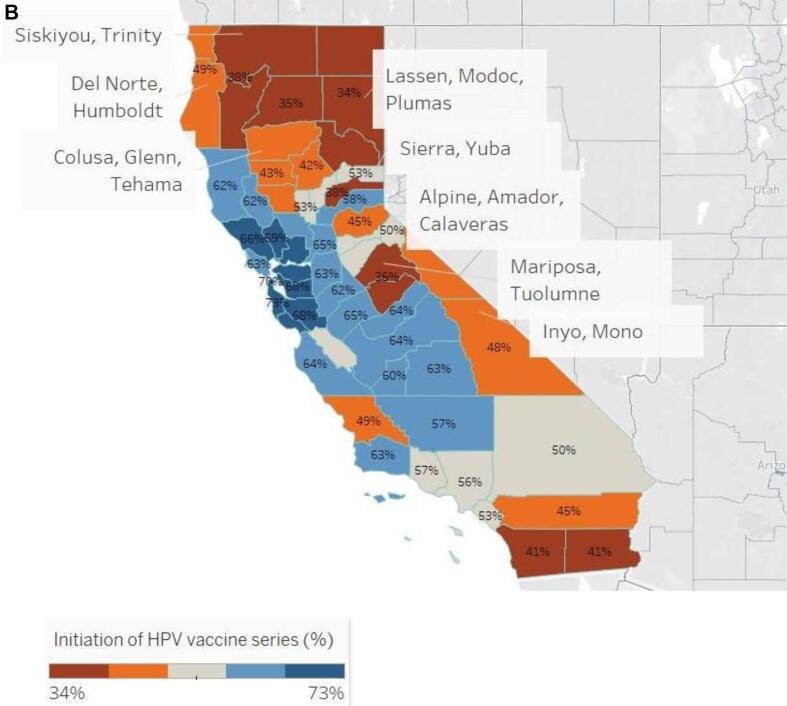
Human Papillomavirus vaccine series initiation proportion by county, California, 2019, California Immunization Registry.

**Fig. 1C:**
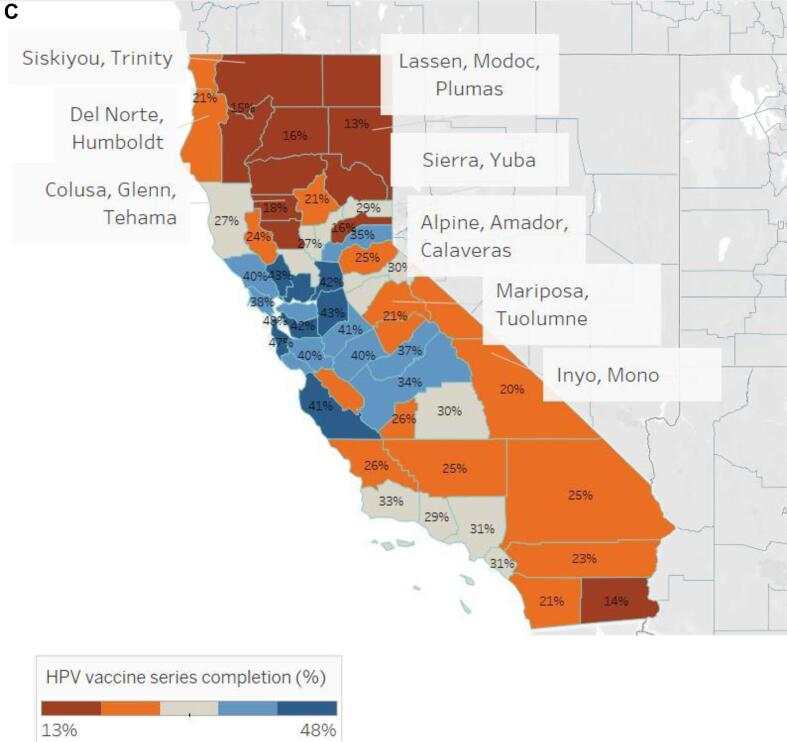
Human Papillomavirus vaccine series completion proportion by county, California, 2019, California Immunization Registry.

## 4 Discussion

Since the study was conceived and conducted, the California State Legislature passed Assembly Bill-1797 in August 2022 that will be effective January 1, 2023. With this Bill, all providers in California will be mandated to report immunizations they administer as well as the patient’s race and ethnicity into CAIR or Healthy Futures. (Weber et al., 2022).

This study compared California HPV vaccination estimates using three sources of immunization data. For California’s 13-year-olds, we observed substantial variation in the estimates of HPV vaccination status among the different immunization reporting systems. For California’s 13-year-olds, HPV vaccination rates varied across the databases with Medi-Cal managed care at 45 % completion, commercial HMO members at 50 %, and CAIR at 28 %. While the private and commercial plan estimates are consistent with NIS-Teen’s 2018 national survey estimate for 13-year-olds (40 %; 95 % CI 37.0–42.9 %) as well as NIS-Teen’s Californian 13- to 17-year-olds (52.6 %; 95 % CI 45.4–59.8 %), CAIR’s immunization data are discordant with these other sources.

Each of these data sources is vital in helping understand HPV vaccination uptake in California. While the NIS-Teen is the most used system to monitor vaccine coverage in the United States, there are limitations at the state level including larger margins of error compared to the national reports and systematic errors of non-response bias. (Wolter et al., 2017, Centers for Disease Control and Prevention National Center for Immunization and Respiratory Diseases, 2020) CAIR differs from NIS-Teen given CAIR is a population-based registry relying on provider reporting. At the time of this study, though, CAIR was limited by lack of mandated reporting. (California State Board of Pharmacy, 2022, Centers for Disease Control and Prevention, 2022) This is likely the main factor contributing to lower reported HPV vaccination rates by this system. The final source of data was gathered from Medi-Cal and California HMO reports. These reports included mixed methods of acquiring vaccine coverage through both random sampling as well as claims, encounters, and supplement EHR data. (Quality Measures & Reporting, 2022, Integrated Healthcare Association, 2021, National Committee for Quality Assurance, 2022). Together, NIS-Teen, CAIR, and health plan and provider group reports were triangulated to provide more accurate trends of HPV vaccination in California’s 13-year-old population.

As a result of Medi-Cal and HMO reporting methods, they may reflect up to 62 % of all 13-year-olds in the State. CAIR has the potential to describe 100 % of the State’s adolescents, but it is evident that CAIR’s lack of standard reporting protocol is impacting the completeness of the reported immunization rates. Examples of single-system provider reporting include Michigan and New York’s immunization information systems. They both require all providers to report their immunizations to the state registry. (Michigan Care Improvement Registry, 2022, New York State, 2022) These populous states serve as a good model and solution for California’s registry.

Similar to national trends, this study also found that regions with significantly lower initiation rates were in rural territories compared to urban areas. (Elam-Evans et al., 2020, Crosby et al., 2011) CAIR records show that San Francisco and San Mateo County have the highest HPV vaccine series completion at 48 % and 49 %, respectively, and Imperial, Lassen, Modoc, and Plumas Counties have the lowest series completion at about 13 %. While this may be due to disproportionate reporting across counties that would account for disproportionate uptake in the HPV vaccine, the initiation and completion in these regions follows the pattern where rural California counties having a higher burden of HPV-related cancer. (Gillette-Walch et al., 2021) Rural counties continue to have various limitations that lead to a decrease in utilization of preventative services. National and state studies have found that limitations in rural areas include decreased number of primary care providers, financial constraints, poor transportation, and fewer access points for vaccination. (Bull et al., 2001, Bouldin et al., 2018, Zhai et al., 2020, Boyd et al., 2018) Further investigation is needed to assess the relationship between those factors and HPV vaccine coverage in rural counties of California. Such studies could help inform interventions aimed to address the increased vulnerability to HPV-related cancers in these regions.

Despite differences in data sources, it is evident that no county or health system in California achieved the U.S. Department of Health and Human Services Healthy People’s recommendation that 80 % of the country’s adolescents be fully immunized against HPV by their 13th birthday. (People, 2030) While the gap is narrowing between male and female HPV initiation and completion rates according to both NIS-Teen and CAIR findings, there remain missed opportunities for vaccination with the NIS-Teen estimates of HPV vaccination series completion peaking at 51.4 % and 61.5 %, respectively, for the study years. (Walker et al., 2019) While this study did not investigate contributing factors in California’s HPV vaccination patterns, decreased awareness of the HPV vaccine^41–43^, lack of school entry requirements, (Ko et al., 2020) parental refusal based on informational, social, or cultural factors, (Wong et al., 2021, Hirth et al., 2019, Brewer et al., 2017, Szilagyi et al., 2020) and lack of standard provider recommendations (Kashani et al., 2019, Dempsey and O’Leary, 2018) have been noted as influences in decreased uptake across the United States. Future studies should elucidate patterns for HPV vaccine hesitancy and refusal in California, by region and population, to improve both initiation and completion rates.

There are limitations to this study. CAIR estimates are impacted by individuals who no longer participate in the immunization information system, or who move out of the state, which inflates the denominator and gives an underrepresentation of true vaccination. To mitigate this inflation, if a child had fewer than two vaccine doses recorded, they were excluded from the analysis. This method could inadvertently exclude unvaccinated children. Additionally, we did not collect initiation rates for the years 2018 and 2019 for Medi-Cal and commercial insurances plans, and completion rates were not gathered for the year 2019 for Medi-Cal and commercial insurances plans, preventing year to year comparisons of the data sources. With both sources, we also are not accounting for patients who are uninsured. Further, even with NIS-Teen as the comparison database, it is not a gold standard since estimates are made based on a few hundred teens within the state. Although there is no gold standard for direct comparison, we were still able to report and compare vaccination patterns for the California databases. Lastly, NIS-Teen does not report on state data for 13-year-olds alone. Given this lack of data, we were unable to do a direct comparison of NIS-Teen estimates for California compared to the other immunization sources for this age group.

## 5 Conclusion

It is important to evaluate the differences of the three HPV vaccination reporting sources in California to estimate current vaccination rates and to understand current patterns throughout the State. Our results suggest that the CAIR database has a lower proportion of HPV vaccination completion among California’s 13-year-olds when compared to commercial and Medi-Cal insurance plans due to differences in assessment of vaccine uptake. Where CAIR currently relies on voluntary provider reporting, the other sources have standardized procedures and sampling. Importantly, California’s adolescent HPV vaccination rates are stabilizing far below Healthy (People, 2030) national goal of 80 % vaccination, and there are lower initiation and completion rates in rural counties. California must follow models of other large states that have a comprehensive immunization registry that includes HPV immunization records for all adolescents within the State. With new mandatory vaccination reporting in 2023 throughout California, this paper will serve as a comparator for future, similar studies of various sources of HPV vaccination rates. Overall, this paper underscores the current similarities and differences of the three databases, and these must be acknowledged when they are being cited as evidence for state and county-wide vaccination policy.

## 6 Future directions

The findings of this study documenting less than 50 % HPV series competition for 13-year-olds ought to inform public health planning. Future work should be twofold: first, on identifying missed opportunities for HPV vaccinations and how to increase HPV vaccination across the state, and second, on investigating the impact of new provider reporting laws on vaccination rates across the three CAIR regions. Solutions for improving HPV vaccination series initiation and completion should ultimately be focused with consideration for regional and health system-level factors. California must prioritize and incentivize vaccine improvements to protect our youth and population from HPV-related cancers.

## 7 Financial disclosure

No financial disclosures were reported by the authors of this paper.

## CRediT authorship contribution statement

**Brooke R. Warren:** Writing – original draft. **Hilary Gillette-Walch:** Data curation, Methodology, Formal analysis. **Jaime Adler:** Methodology, Writing – review & editing. **Raquel Arias:** Methodology, Writing – review & editing. **Jeffrey D. Klausner:** Conceptualization, Writing – review & editing. **Kimlin T. Ashing:** Writing – review & editing. **Alessandro Villa:** Supervision, Conceptualization, Writing – review & editing.

## Declaration of Competing Interest

The authors declare that they have no known competing financial interests or personal relationships that could have appeared to influence the work reported in this paper.

## Figures and Tables

**Table A3:** HMO commercial plans, Integrated Healthcare Association.

**HMO Insurance Plans**
Kaiser-Permanente-Northern California
Kaiser-Permanente-Southern California
CIGNA
Western Health Advantage
Anthem Blue Cross
Blue Shield of California
Sharp Health Plan
UnitedHealthcare of California
Aetna Health of California, Inc.
Health Net of California

## Data Availability

Data will be made available on request.

## Appendix

Table A3.
